# Integrating nano crystal sensor with explainable deep learning for nutrients and microplastic-toxicity detection

**DOI:** 10.1038/s41598-026-51368-3

**Published:** 2026-05-14

**Authors:** Aya Magdy, Seham Abd-Elsamee, Doaa A. Altantawy

**Affiliations:** Electronics and Communications Engineering Department, Faculty of Engineering, Mansoura University, 60 El-Gomhoria Street, Mansoura, 35516 Egypt

**Keywords:** Sustainable agriculture, PhC sensor, Deep learning, Explainable AI (XAI), SHAP and LIME, Engineering, Environmental sciences, Optics and photonics

## Abstract

This work proposes a simulation-based photonic–AI sensing framework for soil nutrient and microplastic detection. This framework integrates a 2D dual-ring cavity photonic crystal (PhC) sensor with a Deep & Cross Network model (DCN). The PhC sensor demonstrates strong optical confinement and spectral selectivity, achieving quality factors up to 18,244, with resonance wavelengths spanning 1528.5–1824.4 nm, high sensitivity as 631 nm/RIU for nutrients and 432 nm/RIU for Low density polyethylene (LDPE) microplastic detection. The PhC sensor further achieves figures of merit up to 3440 RIU⁻¹ and detection limits as low as 3 × 10⁻⁵ RIU, and stable operation under fabrication tolerances and temperature variations. The proposed DCN architecture effectively analyzes the resultant spectral responses of the different soil elements and contaminants. It precisely captures nonlinear spectral patterns without relying on refractive index as an explicit feature, avoiding classification ambiguity at similar concentration levels of soil elements. The introduced DCN model achieves high classification/identification performance of 99.87% accuracy and near-perfect precision, recall, and F1-score. For explainability, SHAP and LIME are employed to quantify spectral feature contributions and explain individual predictions. This explainable AI (XAI) analysis confirms the physical relevance of dominant spectral features used for decision-making. The performed Fabrication tolerance and temperature analyses confirm stable operation within practical limits. Although experimental validation is beyond the scope of this study, the compact sensor design and lightweight inference model support future hardware integration. These results demonstrate the potential of physics-based photonic sensing combined with explainable AI for intelligent soil monitoring.

## Introduction

The agricultural sector plays an important role in the development of a country’s economy^1^. The rapid increase in the world population to 8.9 billion by 2050 will lead to even higher demand for agricultural products^2^. Therefore, there is a growing need for accurate soil analysis for sustainable and environmentally friendly farming methods^3^. Macronutrients and micronutrients that affect plant and productivity such as Nitrogen (N), Potassium (K), Phosphorus (P), Magnesium (Mg), Calcium (Ca), Iron (Fe), Zinc (Zn), Copper (Cu), Sulfur (S) and Boron (B) need to be controlled for better plant performance^4^. Furthermore, Microplastics (MPs) – synthetic polymer particles usually between 50 nm and 5 mm in size – pose a growing threat to soil and its health^5^.

These particles originate either as primary particles from manufactured microfibers and microparticles (e.g., in textiles, medicines, and personal care products) or as secondary particles formed from large-scale plastic materials produced by natural weathering, mechanical abrasion, biological reactions, or human activity^6^. They are difficult to remove from extensive and open systems^7^ and can affect soil structure, reduce water retention, and alter microbial populations, posing a risk to plant health and food safety^8,9^. Due to their low density, MPs are easily carried by the wind and transported to different locations^10^. In clay-rich agricultural soils, especially those containing minerals such as montmorillonite, cracks can act as channels through which MPs migrate to deeper layers, which can affect root systems and soil chemistry^11^.

Some of the traditional soil detection methods are mostly laboratory-based, involving complex sample preparation and time-consuming protocols that can limit productivity^4^. Optical sensing techniques have been developed, such as plasmonic sensors^12^, waveguide sensors^13^, interferometric sensors^14^, and photonic crystal sensors^15^ as a more practical alternative method. Since each soil nutrient and MPs have a specific dielectric constant, electromagnetic analysis holds promise for the specific analysis of plant nutrients despite the complexity of the soil matrix^16^. These optical techniques provide enhanced accuracy, reduced sample requirements, and faster analysis^17^.

Recently, Photonic crystal (PhC)-based sensors have been considered one of the most promising sensing devices due to their superior physical characteristics, such as reflectance/transmittance, compact design, low energy consumption, and a small amount of time and samples. Thus, they can enhance sensitivity, quality factor, and enable precise detection^18^. A PhC is a material with a periodic variation in its refractive index in one (1D)^19^, two (2D)^20^ or in all three (3D)^21^ orthogonal directions. Although 1D is less expensive and simple in fabrication, it has a narrow photonic band gap (PBG) that limits the range of wavelengths that can be sensed^22^. 2D PhCs are easy to fabricate and have more potential. In comparison with 1D and 2D structures, 3D PhCs are more complicated to fabricate and have limited 3D space^23^. Due to periodicity, the light transmission will be zero through a defined range of frequencies that is called the photonic band gap (PBG). When defects disturb this periodicity, it allows the control and manipulation of light^24^. Photonic crystal sensors offer exceptional sensitivity and selectivity for chemical and biological detection, but their design and optimization typically require extensive simulation and expert knowledge. Therefore, different AI-based frameworks integrate machine learning/deep learning with photonic-crystal modeling to accelerate device design^25^, enhance sensing performance^26^, and enable rapid parameter optimization^27^. Hence, this study presents a fully simulation-based photonic–AI sensing framework with the following contributions:

1. ***Design of a novel photonic crystal–based sensor for soil analysis***.
  We propose a photonic crystal structure specifically engineered to detect key soil elements and plastic-related contaminants. The sensor design enhances sensitivity by optimizing the photonic bandgap response to variations in refractive index caused by different contaminants.
2. ***Demonstration of optical detection for both elemental composition and microplastic contaminants.***
  The proposed sensor provides a dual-purpose detection mechanism capable of identifying chemical elements and plastic-derived pollutants within soil samples, enabling a more comprehensive environmental monitoring platform.
3. ***Development of a Deep Cross Network–based classification model for detected signatures***.
  We implement a DCN deep learning architecture to classify the optical signatures generated by the photonic crystal sensor. This model effectively learns high-order interactions between spectral features, improving identification accuracy over conventional neural networks.
4. ***Creation of an integrated photonic–AI sensing pipeline.***
  By combining photonic crystal optical detection with a state-of-the-art deep learning classifier, the work introduces an end-to-end sensing framework capable of rapid, automated, and high-precision soil contaminant analysis.
5. ***Performance evaluation.***
  Through numerical simulations and python-based deep learning pipeline, we verify the sensor’s sensitivity and demonstrate that the DCN model achieves superior recognition accuracy for multiple soil elements and plastic contaminants compared with baseline methods.
6. ***Robustness***,*** fabrication feasibility***,*** and deployment considerations.***
  The study includes tolerance and temperature stability analysis, clarifies practical nanofabrication pathways for silicon–germanium cavity realization, and discusses future embedded implementation for real-time inference. This strengthens the translational relevance of the proposed system while clearly defining current simulation-based limitations.

## Background and related work

### Traditional soil nutrient detection

Wet chemical soil extraction is one of the traditional ways of detecting nutrients in the soil. It was utilized to extract substances (e.g., dithionite, oxalate, and pyrophosphate) in identifying pedogenic aluminium (AL) and iron (Fe) species for soil classification. Researchers found that chemical extraction lacks selectivity and completeness, so it is unreliable for different types of soil^28^. Also, microscopic and spectroscopic techniques^29^ have been used to quantify some soil nutrients (e.g., Si, Al, Fe, Ca, K, Mn). But these techniques could not detect other soil nutrients like Cl and Zn, due to their low atomic number and weak X-ray emission signals^30^. Despite being faster than traditional wet chemistry, these techniques are still laboratory-based and not suitable for real-time soil analysis^31^.

### Photonic crystal-based sensing

A lot of research discussed implementing PhCs-based sensors in many aspects (e.g., environmental^4,32^, medical^33–36^ and chemical^37–39^. In the medical sector, a 1D photonic crystal-based sensor was designed to detect blood plasma and cancer cells. The sensor consists of a sample layer between two identical photonic crystals of silica (SiO_2_) and titania (TiO_2_). Performance was evaluated using the transfer matrix method, showing a sensitivity of 71.25 nm/RIU for a sample thickness of 100 nm, which can be increased to 161 nm/RIU by increasing the sample thickness to 300 nm^36^. For chemical investigation, pressure was sensed, a 2D PhC sensor with a square array of silicon rods surrounded by air, and an L3 defect was placed between two waveguides used to detect pressure range between 2 GPa and 7 Gpa. The sensor achieved QF of 772^38^. Besides, a ternary photonic crystal(TPC) has alternate layers of glass, dielectric, and semiconductor material with ethanol solution as a defect layer to detect water concentration in ethanol. Demonstrating a sensitivity of 144.369 nm/RIU^39^. In environmental monitoring, a 1D photonic crystal sensor formed by alternating layers of magnesium fluoride (MgF2) and silicon (Si) with an empty layer in the middle, the defect layer is filled with polluted air. The sensor achieved a sensitivity of 700 nm\RIU, a high QF of 300,000, and a figure of Merit (FoM) of 149,000 RIU^− 1^ in detecting hazardous gases such as CO₂ ^40^. In addition to gas detection, a 2D photonic crystal (PhC) biosensor is utilized to detect bacterial contamination by monitoring changes in the refractive index of water. A hexagonal lattice of silicon rods was designed using RSoft Photonic Suite (CAD) Computer-Aided Design. The sensor includes two waveguides and a defective circular microcavity where bacteria affect light propagation, achieving a high sensitivity of 834.344 nm/RIU^41^. Moreover, a 2D-PhC hexagonal resonator was considered one of the first applications of PhC technology in soil analysis, with dimensions of 11.4 × 9.2 μm. The sensor achieved a high-quality factor (QF) of 228.6 with 100% transmission efficiency at a resonant wavelength of 1600 nm ^4^. Although previous studies^4,41,42^ have applied PhC sensors to detect gases, water quality, and soil conditions, it is still limited. Developing 2D PhC sensors for soil nutrient and microplastics detection plays a vital role in environmental monitoring. Combining such sensors with deep learning can make detection faster, more accurate, and smarter for real applications^33,34^.

### AI-based photonic crystal sensors

PhCs often require computationally expensive numerical solvers, such as the plane-wave expansion (PWE)^43^, finite-difference time-domain (FDTD) methods^44^, and Finite Element methods^45^. Hence, recent studies tend to employ machine learning (ML) and deep learning (DL) techniques in the design, analysis, and optimization of photonic crystal (PhC) structures. ML-based surrogate models and inverse-design strategies have emerged as powerful alternatives. Existing studies can be roughly categorized into three major fields: (1) forward modelling^46,47^, (2) inverse design^48^, and (3) sensor design^27^.

In forward modeling, ML predicts optical properties directly derived from structural parameters. This is a fast and very accurate alternative to numerical solvers. ML has been used to improve prediction accuracy and address limitations of human tuning in photonic crystal structures^49^. For instance, a machine learning–assisted 2D photonic crystal biosensor was able to accurately detect the resonant wavelength for various types of cancer cells, such as HeLa, PC-12, MDA-MB-231, MCF-7, and Jurat, using silicon-on-insulator (SOI) technology^46^. The structure contained three cavities: two circular (C1, C3) and one central hexagonal (C2), where the analyte is positioned, achieving a high-quality factor of 14,782, a sensitivity up to 55 nm/RIU, an extremely narrow spectral linewidth of 0.1 nm, and a prediction accuracy of 99%. Similarly, a photonic crystal fiber (PCF) with two rings of air holes arranged in a hexagonal pattern with four gold nanowires is placed symmetrically in the structure to create (SPR) surface plasmon resonance. Artificial neural networks (ANNs) are used to predict key sensor parameters like confinement loss and sensitivity, eliminating the need for repeated and time-consuming numerical simulations. This hybrid SPR–PCF–ML achieved a sensitivity ranging from 2000 to 18,000 nm/RIU and a resolution of 5.56 × 10⁻⁶ RIU^47^. On the other hand, studies on inverse design concentrate on deriving analyte characteristics from optical outputs. A deep learning algorithm has been developed to predict the refractive index (RI) of analytes in an open channel plasmonic sensor^48^. The deep learning models (ANN) Artificial Neural Network, (CNN) Convolutional Neural Network, and (LSTM) Long Short Term Memory, use data generated from simulations of the open channel plasmonic sensor (e.g., wavelength, confinement loss) and predict the analyte’s refractive index (discrete RI classes (1.33–1.40)), which changes when a new sample is detected. Furthermore, the sensor design category focuses on leveraging AI algorithms to optimize the design parameters of photonic crystal sensors. According to this category, a multi-objective gray wolf optimizer was utilized to find the optimized parameter values for a PhC liquid sensor without human interference^27^. Furthermore, the results indicate that the proposed framework can design any kind of PhC sensor.

In this work, we introduce Photonic–AI integrated sensing system. It combines a physics-based photonic crystal (PhC) sensor design with a data-driven deep learning classifier. It falls under hybrid physical–AI systems. The photonic structure encodes material properties into spectral responses (physics layer), and the deep-cross network performs nonlinear discrimination for the detected soil component (AI layer). This framework faces Bi-perspective challenges. From a photonic perspective, the proposed sensor should achieve.

- **High sensitivity to refractive index changes**: Soil components with different dielectric properties induce subtle shifts in the resonance spectrum. PhC structures amplify these variations through strong light–matter interaction^50^.
- **Spectral fingerprint generation**: Even when components exhibit similar bulk properties, their interaction with the photonic structure produces distinguishable multi-feature spectral signatures (e.g., sensitivity and quality factor).

some soil components exhibit very close optical parameters, particularly at certain concentration ranges. In these cases, the resulting spectra become highly similar and may not be linearly separable using conventional threshold-based or shallow learning methods From an AI perspective. Hence, from AI-based perspective, the challenges of the proposed framework can be summarized as.

- **Explicit feature interaction modeling**: DCNs are particularly effective in capturing higher-order feature interactions. In our case, subtle relationships between spectral features (e.g., correlations between multiple resonance peaks) are crucial for distinguishing closely related soil components.
- **Improved discrimination in near-degenerate cases**: When two soil components produce overlapping spectral shifts at specific concentrations, discrimination depends on nonlinear combinations of spectral features rather than single-peak analysis.
- **Reduced manual feature engineering**: Instead of relying on handcrafted spectral descriptors, the DCN automatically learns relevant feature crosses.

## The proposed sensing system

**Fig. 1:**
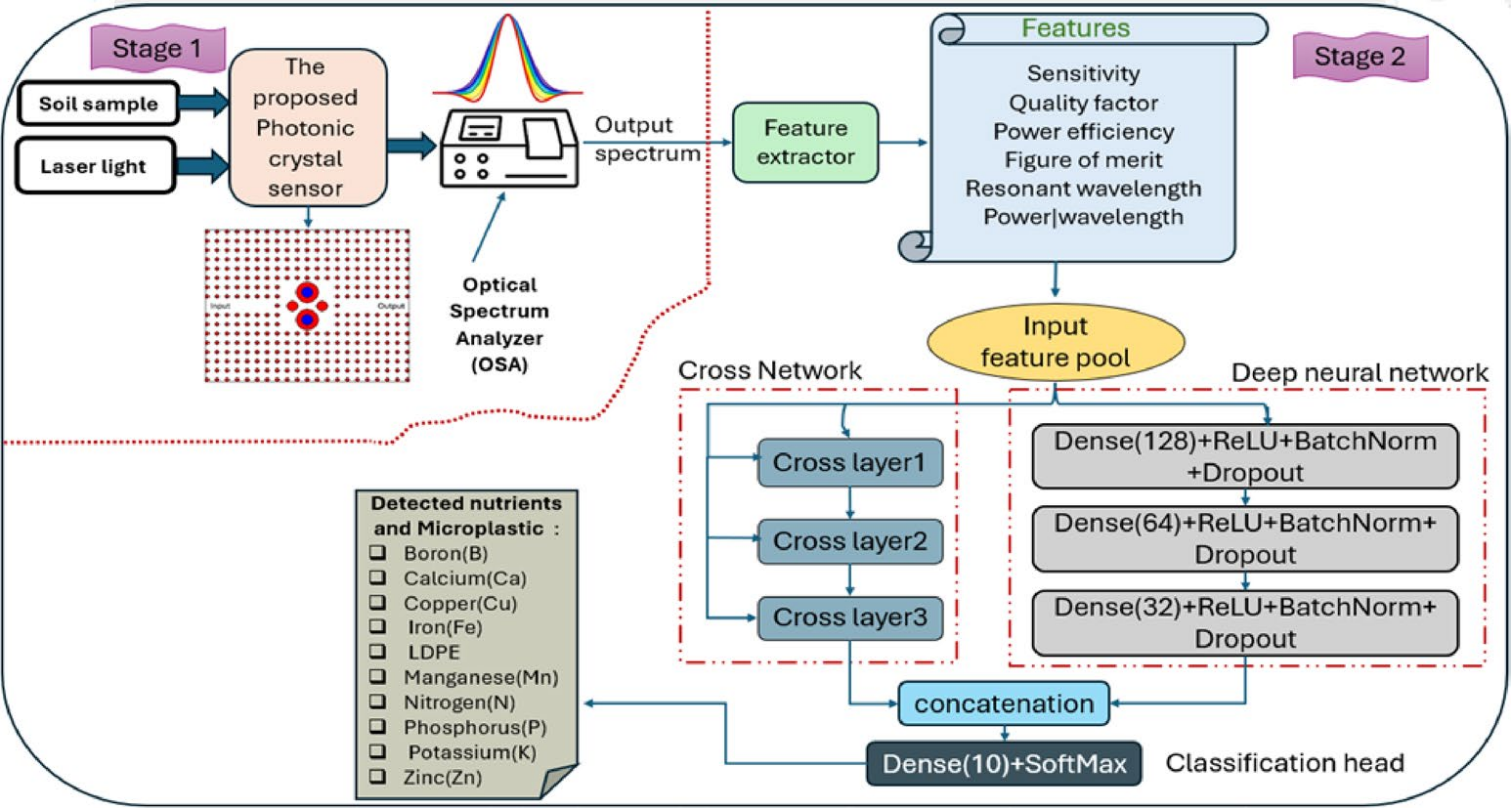
Block diagram of the proposed two stages nutrient and microplastic detection system integrating a photonic crystal sensor with a deep learning-based classification model.

The proposed sensing system consists of two main stages, as shown in Fig. 1. First, a laser passes through a special design 2D-dual-ring cavity PhC sensor, which analyzes the samples and produces an optical spectrum. Important optical features like refractive index, sensitivity, and resonance wavelength are then extracted. In the second stage, these features are processed by a deep learning model that uses cross-network and dense layers to identify specific nutrients and microplastics accurately. This approach combines precise optical sensing with intelligent data analysis for efficient material detection.

### Stage 1: The proposed PhC sensor

**Fig. 2:**
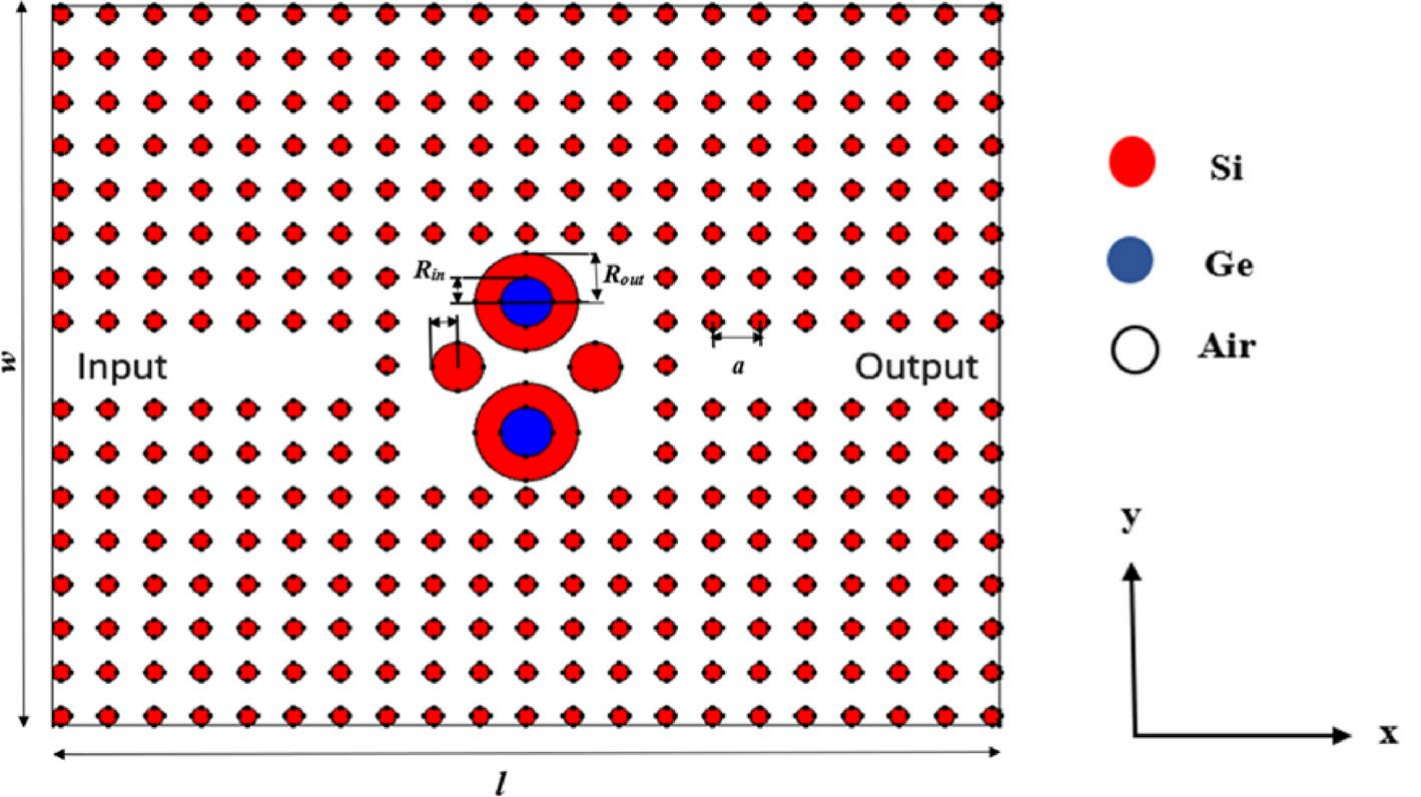
Schematic of the proposed 2D dual-ring photonic crystal sensor, where silicon rods (red), germanium rods (blue), and air holes (white) form the periodic lattice. The soil analyte is assumed to occupy the air-hole background region surrounding the defect cavities.

A 2D-dual-ring cavity PhC sensor, with the dimensions of 11 μm x 8.85 μm, was designed using a square lattice arrangement (21 × 17) of silicon circular rods with a refractive index of 3.4 in an air background, with a lattice constant (*a*) of 540 nm and a rod radius (*r*) of 108 nm. The proposed sensor is designed such that the analyte to be detected infiltrates the air-hole background of the photonic crystal lattice surrounding the defect cavities, enabling refractive-index modulation of the resonant modes. At the center of the sensor, a defect is introduced. This defect consists of two circular cavities: an outer silicon cavity with a radius (*R*_*out*_) of 600 nm and an inner Germanium (Ge) cavity with a radius of 300 nm. Germanium was selected for its higher refractive index of 4 to enhance the optical confinement within the defect. Two symmetric circular Silicon rods with a radius (*R*_*in*_) of 300 nm are adjacent to the defect, to facilitate directional light coupling as shown in Fig. 2. The optimized geometric parameters of the proposed nano photonic crystals are listed in Table 1, based on the analysis presented in the results and discussion section. The proposed sensor is designed to hold the analyte (that needs to be detected) in its background, enabling accurate sensing based on the shift in the resonance wavelength because of changes in the analyte’s refractive index from 1.0003 to 1.8600 for soil nutrients detection and from 2.2 to 2.5 for microplastics. The simulation has been carried out using the 2D Finite Element Method (FEM)^45^.

**Table 1:** Geometric parameters of the proposed sensor.

Geometric Parameters	Dimensions
Length of PhC platform (*L*)	11 μm
Width of PhC platform (*w*)	8.85 μm
Lattice constant (*a*)	540 nm
Radius of Outer Si cavity *(R*_*out*_)	600 nm
Radius of inner Ge cavity (*R*_*in*_)	300 nm

One of the most important factors in designing photonic crystal sensors is the quality factor (QF). It is the ratio of the resonant wavelength to the full width of half maximum^51^ QF can be calculated using Eq. (1):

1 $$\:QF=\frac{{\lambda\:}_{0}}{\varDelta\:{\lambda\:}_{FWHM}}\:$$

Where$\:\:{\lambda\:}_{0}$ is the resonant wavelength, and$\:\:{\Delta\:}{\lambda\:}_{FWHM}$ is the full width at half maximum spectrum. Sensitivity (S) refers to the ability of the sensor to differentiate between different analytes^51^ can be calculated using Eq. (2):

2 $$\:S=\varDelta\:\lambda\:/\varDelta\:n\:\:\:\:$$

Where $\:\varDelta\:\lambda\:$ is the change in resonance wavelength, and $\:\varDelta\:n$ is the change in the refractive index between the reference medium and the analyte refractive index. The figure of merit (FoM) is another important parameter; it detects the smallest change in the optical sensor and is proportional to sensitivity (S) and quality factor (QF)^51^. FoM can be calculated using Eq. (3):

3 $$\:FoM\:=\:\frac{S}{\left(\varDelta\:{\lambda\:}_{FWHM}\right)}\:\:\:$$

Where *S* is the sensitivity and, $\:\varDelta\:{\lambda\:}_{FWHM}$ is full the width at half maximum spectrum. Power efficiency (η) indicates how much energy is used to do the work, compared to the amount of wasted energy^51^ can be calculated using Eq. (4):

4 $$\:\eta\:=\:\frac{{P}_{out}}{{P}_{in}}\:x\:100\:$$

Where, $\:{P}_{out}$ and $\:{P}_{in}$ are the input signal power and the output signal power. The detection limit (DoL) is defined as the smallest analyte concentration that can be detected and depends directly on the sensitivity (S) and quality factor (QF)^33^. Therefore, optimizing the device geometry to enhance S and QF leads to a reduced DoL.In this work, Eq. (5) serves as a unified performance metric guiding the geometric tuning of the dual-ring cavity to achieve improved sensing resolution. DoL can be calculated using Eq. (5):

5 $$\:DoL=\frac{{\lambda\:}_{0}}{10xSxQF}$$

### Stage 2: The proposed deep nutrient and micro-plastic toxicity detection

Now, after the proposed photonic crystal sensor performs the physical detection of nutrients and plastic contaminants, the proposed deep learning model interprets the spectral signatures to accurately identify the detected species. Hence, this stage will be divided into subsections the first is about how the dataset is collected and the second is about the proposed explainable deep learning model.

#### Data collection and problem formulation

The dataset was constructed by modeling the response of the designed photonic crystal (PhC) soil sensor under varying refractive indices corresponding to different soil nutrients and concentration levels. The nutrients considered are Boron (B), Calcium (Ca), Copper (Cu), Iron (Fe), LDPE, Manganese (Mn), Nitrogen (N), Phosphorus (P), Potassium (K), and Zinc (Zn). Each nutrient exhibits a characteristic refractive index range, which varies with concentration^52^. The changes in concentration level usually cause small changes in the nutrient refractive index^52^. In each simulation trial performed, we get one simulated spectral response of the PhC sensor under a specific nutrient type and concentration. In addition, the following features are gathered: power versus wavelength (P versus λ), resonance wavelength (λ_peak), power efficiency η (%), quality factor (QF), sensitivity S (nm/RIU), figure of merit FoM (RIU⁻¹), and the structural configuration parameters of the sensor: Lattice constant a, Width $\:w$, Length $\:l$, Outer radius $\:{R}_{out}$, and Inner radius $\:{R}_{in}$.

Now all the performed simulations for each nutrient under varying refractive index conditions, because of the concentration levels, are stored in a CSV file. Each resultant spectrum is represented by multiple wavelength sampling points, where every row in the dataset corresponds to a specific wavelength (λ) and its associated power (P), while maintaining the same structural parameters and resonance characteristics such as peak wavelength (λ_peak), transmission efficiency (η), quality factor (QF), sensitivity (S, nm/RIU), and figure of merit (FoM, RIU⁻¹) corresponding to this spectrum. This representation preserves both the fine spectral shape information and the derived sensing performance metrics. Accordingly, the proposed Deep Cross Network becomes able to learn subtle nonlinear feature interactions, particularly in cases where different nutrients exhibit very similar refractive indices and closely overlapping resonance peaks at certain concentrations. This task is formulated as a supervised multi-.

class classification problem, i.e., the DCN model is trained using input–output pairs (features $\:{X}_{0}$ and correct labels $\:Y$). In the setting, the extracted sensor features $\:{X}_{0}\:\in\:{\mathbb{R}}^{3750\times\:12}$, such as wavelength, transmission intensity, resonance peak, Q-factor, and sensitivity, are mapped to one of the predefined ten nutrient categories $\:Y\in\:{\mathbb{R}}^{3750\times\:1}$, (e.g., Nitrogen, Phosphorus, Potassium, etc.). Before this mapping or training process is performed, a feature reduction process is performed to improve model performance and robustness. Feature reduction removes weakly informative variables, prevents overfitting, especially given the relatively small and structured dataset, and improves computational efficiency and model generalization. Mutual information MI is used for feature reduction. MI measures the dependency between each feature ​$\:{{x}_{0}}_{i}$ and the target variable $\:Y$ as described in Eq. (6):

6 $$\:I\left({X}_{0};\:Y\right)=\sum\:_{{{x}_{0}\in\:X}_{0}}{\sum\:}_{y\in\:Y}p({x}_{0},y)log\left(\frac{p({x}_{0},y)}{p\left({x}_{0}\right)p\left(y\right)}\right)$$

**Fig. 3:**
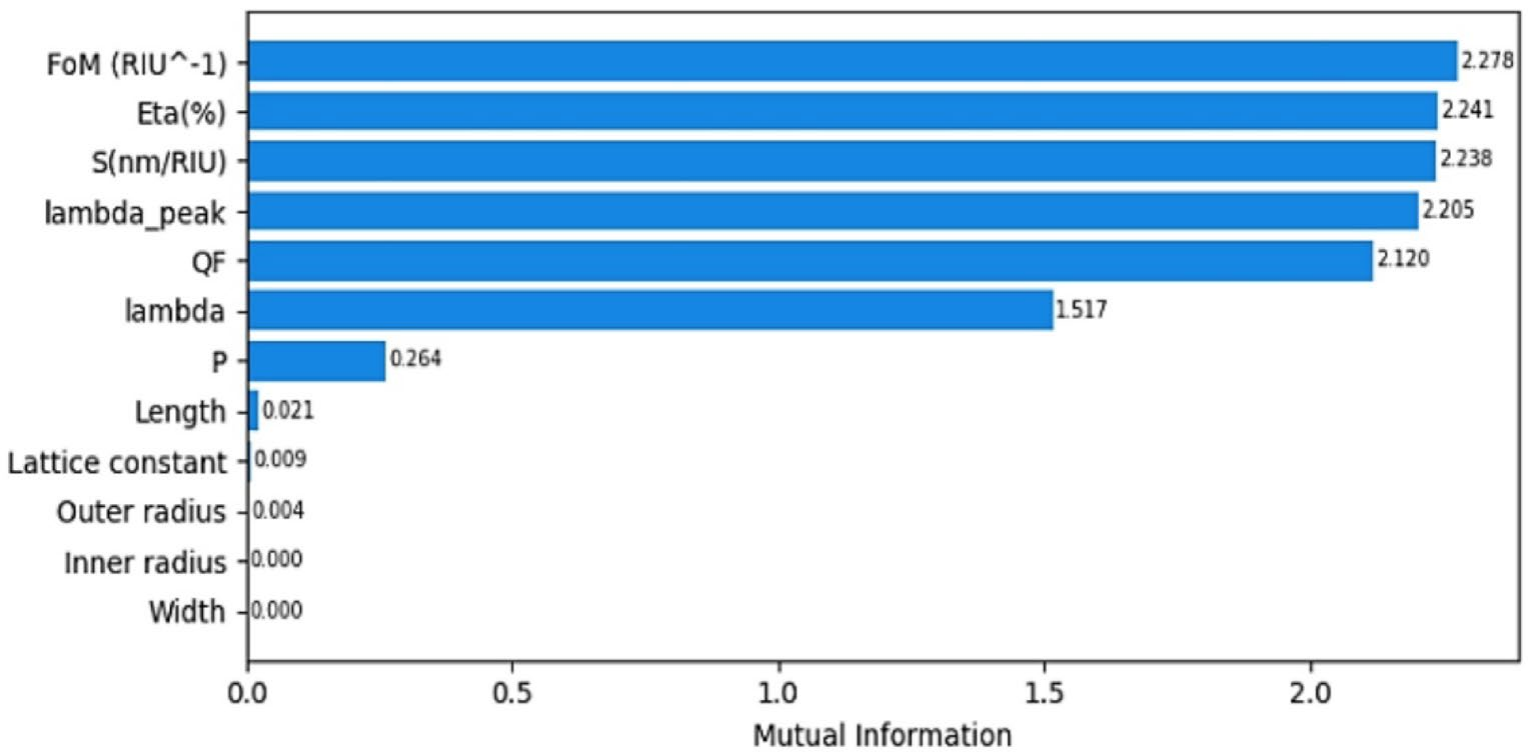
All features importance according to mutual information.

Figure 3 indicates the mutual information feature importance. As indicated, only the seven most informative features (FoM, η, S, λ_peak, QF, λ, and P) were retained, while the remaining features exhibited negligible contribution and were discarded. Consequently, the final input to the DCN model is reduced to $\:{X}_{0}\:\in\:{\mathbb{R}}^{3750\times\:7}$.

**Fig. 4:**
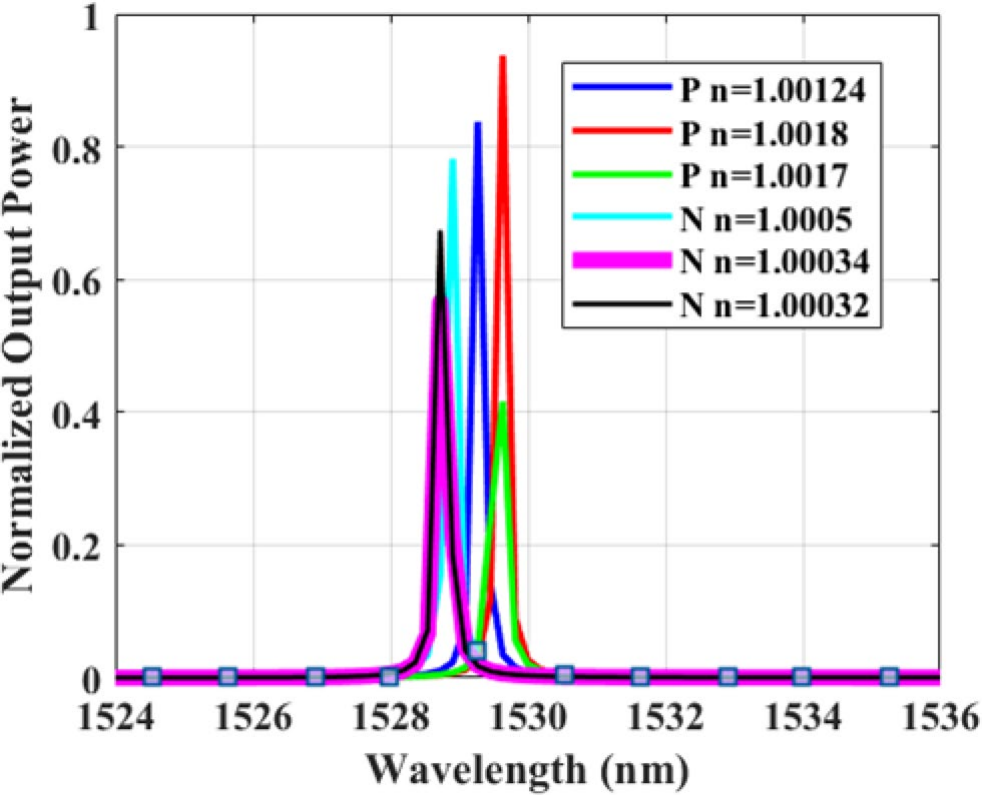
Normalized Transmission spectrum vs. wavelength (nm) for Nitrogen (N) and Phosphorus (P) at three different concentrations of each nutrient.

Figure 4 indicates the spectrum of Nitrogen (N) and Phosphorus (P), two of the most important nutrients, each at three different concentrations. The six spectra were selected as representative examples from the dataset to illustrate the resonance characteristics of different nutrient classes, as well as the effect of refractive index variation on peak shift. Table 2 shows the corresponding numerical values with only three instances of P versus λ.

*N* and *P* are selected as representatives of the targeted soil elements to assess what happens at different concentrations that cause only a very small change in RI. Small variations in the refractive index (Δn ≈ 10⁻⁴–10⁻³) primarily cause a measurable shift in the resonance wavelength, while preserving the overall spectral shape. The sensitivity remains nearly constant if the response is linear, whereas the quality factor may fluctuate slightly due to minor changes in field confinement and loss. The figure of merit can vary more noticeably since it depends on both sensitivity and peak linewidth. Overall, such small refractive index changes confirm stable, linear sensor operation with high resolution and reliable performance.

#### The proposed deep model

To effectively capture the complex relationships embedded in the output spectrum of the proposed photonic crystal (PhC) sensor and its corresponding refractive index of the targeted component, we incorporate the Deep Cross Network v2 (DCN-v2) architecture^52,53^. DCN-v2 provides a powerful mechanism for learning explicit and implicit feature interactions. Hence, it is well suited for our dataset where correlations among features play a critical role in classification accuracy. Deep & Cross Network v2 (DCN-V2) extends the original Deep & Cross Network^52^. The new DCN-V2 introduces a more expressive cross-operation based on full matrix transformations and a low-rank parametrization that reduces computational burden without compromising representational power. DCN-V2 exists in two configurations: parallel and stacked^53^. In this work, the proposed model operates in a parallel dual-tower configuration, in which an explicit cross tower runs alongside a standard deep network, and their outputs are fused before final prediction, see stage 2 in Fig. 1.

*A. Input Representation*.

Let $\:{X}_{0}\in\:{\mathbb{R}}^{d}\:$denote the model’s input vector, formed by concatenating the inserted dense numerical features, i.e., refractive index and the extracted features from the output spectrum. The goal of DCN-V2 is to learn a predictive function $\:Y=F\left({X}_{0}\right)$ that leverages structured feature interactions.

*B. Matrix-Based Cross Layer*.

The $\:{l}^{th}$ cross layer in DCN-V2 computes an explicit bounded-degree polynomial interaction between ​$\:{X}_{0}$ and the intermediate representation $\:{X}_{l}$​. Unlike DCN-V1, which uses a rank-1 (outer-product) structure^53^, DCN-V2 employs a full linear transformation:

7 $$\:{X}_{l+1}=\text{}{X}_{0}{\left({W}_{l}{X}_{l}+{b}_{l}\right)}^{T}+{X}_{l}$$

Where $\:{W}_{l}\in\:{\mathbb{R}}^{d\times\:d}\:$is a learnable weighting matrix, $\:{b}_{l}\:\in\:{\mathbb{R}}^{d}$ denotes the bias vector, and the residual term$\:{X}_{l}$​ preserves identity mappings.

The formulation in Eq. (7) produces a directional “cross” interaction in which each output dimension depends on a weighted combination of all pairwise interactions between the elements of $\:{X}_{0}$​ and $\:{X}_{l}$​. Expanding the expression clarifies the interaction structure:

8 $$\:{X}_{l+1}={\left({X}_{0}{X}_{l}\right)}^{T}{{W}_{l}}^{T}+{X}_{0}{{b}_{l}}^{T}+{X}_{l}$$

**Table 2:** The numerical values of the extracted features at different concentrations for both Nitrogen (N) and Phosphorus (P).

**Y**		**X**_**o**_												
**Target Name**	**n**	**Lattice constant**	**Width**	**Length**	**Outer radius**	**Inner radius**	**λ_peak**		**λ**	**P**	**η (%)**	**QF**	**S (nm/RIU)**	FoM (RIU^-1^)
Nitrogen (N)	1.00032	540	11	8.85	600	300	1528.7		1528.8	0.62	67.4	5095	625	2083
Nitrogen (N)	1.00032	540	11	8.85	600	300	1528.7		1528.6	0.43	67.4	5095	625	2083
Nitrogen (N)	1.00032	540	11	8.85	600	300	1528.7		1528.9	35	67.4	5095	625	2083
Nitrogen (N)	1.00050	540	11	8.85	600	300	1528.9		1528.8	0.57	78.2	7645	800	4000
Nitrogen (N)	1.00050	540	11	8.85	600	300	1528.9		1529	0.55	78.2	7645	800	4000
Nitrogen (N)	1.00050	540	11	8.85	600	300	1528.9		1529.1	0.45	78.2	7645	800	4000
Nitrogen (N)	1.00034	540	11	8.85	600	300	1528.7		1528.8	0.50	57.0	5095	588	1960
Nitrogen (N)	1.00034	540	11	8.85	600	600	1528.7		1528.6	0.35	57.0	5095	588	1960
Nitrogen (N)	1.00034	540	11	8.85	600	600	1528.7		1528.9	0.33	57.0	5095	588	1960
Phosphorus(P)	1.00124	540	11	8.85	600	300	1529.3		1529.2	0.73	83.7	7646	645	3225
Phosphorus(P)	1.00124	540	11	8.85	600	300	1529.3		1529.4	0.53	83.7	7646	645	3225
Phosphorus(P)	1.00124	540	11	8.85	600	300	1529.3		1529.1	0.33	83.7	7646	645	3225
Phosphorus(P)	1.00170	540	11	8.85	600	300	1529.6		1529.7	0.37	41.6	5098	647	2157
Phosphorus(P)	1.00170	540	11	8.85	600	300	1529.6		1529.5	0.34	41.6	5098	647	2157
Phosphorus(P)	1.00170	540	11	8.85	600	300	1529.6		1529.4	0.23	41.6	5098	647	2157
Phosphorus(P)	1.00180	540	11	8.85	600	300	1529.6		1529.7	0.82	93.7	7648	611	3055
Phosphorus(P)	1.00180	540	11	8.85	600	300	1529.6		1529.5	0.54	93.7	7648	611	3055
Phosphorus(P)	1.00180	540	11	8.85	600	300	1529.6		1529.8	0.40	93.7	7648	611	3055

Here, the tensor $\:{\left({X}_{0}{X}_{l}\right)}^{T}\in\:{\mathbb{R}}^{d\times\:d}$ encodes all second-order interactions between feature dimensions. Stacking multiple cross layers iteratively composes these interactions, resulting in an explicit feature expansion whose depth is bounded by the number of cross layers. With $\:L\:$layers, the final cross tower representation is $\:{X}_{cross}={X}_{L}$.

*C. Low-Rank Factorization for Scalable Explicit Interactions*.

The full matrix $\:{W}_{l}\:$scales quadratically with input dimension. To control complexity and improve generalization, DCN-V2 decomposes$\:{W}_{l}$​ via a rank-$\:r$ factorization:

9 $$\:{W}_{l}={U}_{l}{{V}_{l}}^{T}$$

$$\:\mathrm{w}\mathrm{h}\mathrm{e}\mathrm{r}\mathrm{e}\:{U}_{l},\:{V}_{l}\in\:{\mathbb{R}}^{d\times\:r}\:\mathrm{a}\mathrm{n}\mathrm{d}\:r\ll\:d$$

Substituting into the cross operator in Eq. (7):

10 $$\:{X}_{l+1}=\text{}{X}_{0}{\left({U}_{l}{{V}_{l}}^{T}{X}_{l}+{b}_{l}\right)}^{T}+{X}_{l}\:\:\:\:$$

Define the two projections:

11 $$\:{Z}_{l}={{V}_{l}}^{T}{X}_{l}\:\:\:\in\:{\mathbb{R}}^{r},\:\:\:\:\:\:{h}_{l}={U}_{l}{Z}_{l}+{b}_{l}\:\:\in\:{\mathbb{R}}^{d}$$

Then the cross layer becomes:

12 $$\:{X}_{l+1}=\text{}{X}_{0}{{h}_{l}}^{T}+{X}_{l}$$

This reveals that DCN-V2 constructs explicit feature interactions through a sequence of linear projections into a low-dimensional interaction space, followed by a reconstruction back to the original dimension. The model thus balances expressiveness and efficiency: interactions remain global across all features, but their parameterization is compressed.

*D. Parallel Dual-Tower Structure (Parallel Form)*.

DCN-V2 is organized in parallel architecture combining a deep implicit interaction tower and a cross explicit interaction tower. Formally:

*Cross Tower (Explicit Interactions)*

13 $$\:{X}_{cross}={\varphi\:}_{L}\left({X}_{0}\right)$$

where $\:{\varphi\:}_{L}$denotes $\:L$ stacked matrix-based cross layers.

*Deep Tower (Implicit Interactions)*.

A standard multilayer perceptron (MLP):

14 $$\:{h}_{1}=\sigma\:\left({W}^{\left(1\right)}{X}_{0}+{b}^{\left(1\right)}\right)$$

15 $$\:{{X}_{deep}=h}_{k}=\sigma\:\left({W}^{\left(k\right)}{h}_{k-1}+{b}^{\left(k\right)}\right)$$

*E. Fusion and Prediction Layer*.

The towers are combined in parallel via concatenation:

16 $$\:{X}_{final}=\left[{X}_{cross}\left|\right|{X}_{deep}\right]$$

and passed to a final classifier head

17 $$\:\widehat{Y}=g({{W}_{out}}^{T}{X}_{final}+{b}_{out})$$

Where $\:g(.)$ is the output SoftMax activation. Table 3 indicates the proposed model summarization for nutrients and contaminants identification.

## Results and discussion

### Stage 1: 2D-dual-ring cavity PhC sensor

**Fig. 5:**
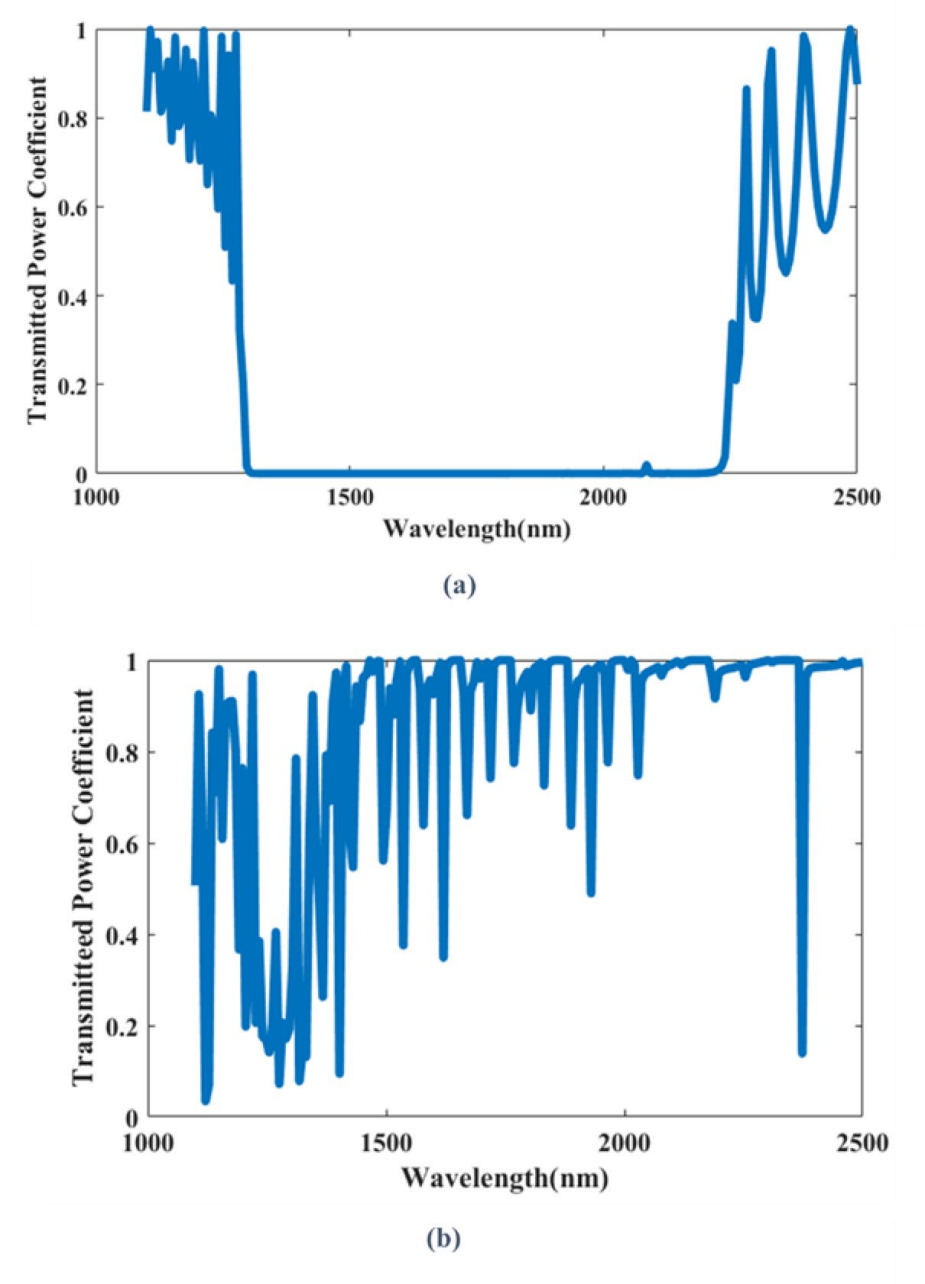
Transmission spectra of the 2D-PhC sensor platform without defects: **(a)** TE mode, **(b)** TM mode.

The evaluation of the transmission spectra of the proposed sensor. The transmission spectra of TE and TM modes are shown in Figs. 5(a), (b), respectively. The TE mode demonstrates a photonic band gap within the range of 1304 nm to 2225 nm. While TM mode does not demonstrate well-defined PBG. It exhibits multiple resonance beaks. So, the TE mode was chosen because of the well-defined PBG.

**Table 3:** Summarization of the employed layers in the proposed DCN-V2 model.

Layer (type)	Output Shape	Param #	Connected to
input (Input Layer)	(None, 7)	0	-
dense (Dense)	(None, 128)	1,024	input[0][0]
batch normalization (Batch Normalization)	(None, 128)	512	dense[0][0]
dense_1 (Dense)	(None, 64)	8,256	batch_normalization[0][0]
batch_normalization_1 (Batch Normalization)	(None, 64)	256	dense_1[0][0]
dropout (Dropout)	(None, 64)	0	batch_normalization_1[0][0]
dense_2 (Dense)	(None, 32)	2,080	dropout[0][0]
batch_normalization_2 (Batch Normalization)	(None, 32)	128	dense_2[0][0]
cross layer (Cross Layer)	(None, 7)	42	input[0][0]
dropout_1 (Dropout)	(None, 32)	0	batch_normalization_2[0][0]
concatenate (Concatenate)	(None, 39)	0	cross layer[0][0], dropout_1[0][0]
dense_3 (Dense)	(None, 10)	400	concatenate[0][0]

Total params: 37,200 (145.32 KB) Non-trainable params: 448 (1.75 KB).

Trainable params: 12,250 (47.85 KB) Optimizer params: 24,502 (95.71 KB).

**Fig. 6:**
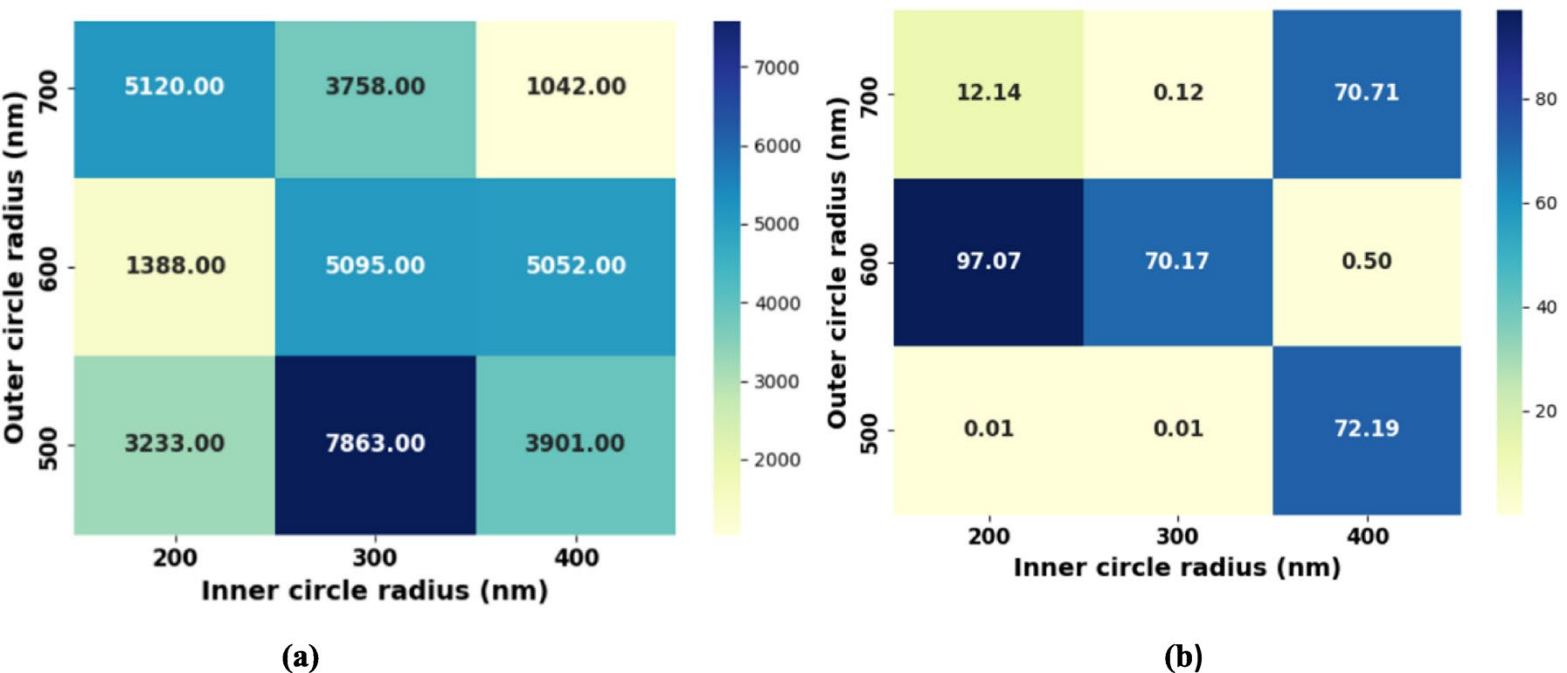
Parametric analysis of the proposed dual-ring cavity sensor as a function of inner and outer circle radii: **(a)** absolute quality factor (QF) values obtained from the geometric parameter sweep, and **(b)** corresponding normalized output power under.

A parametric analysis was performed to determine the optimal geometric dimensions that maximize the quality factor (QF). Figure 6(a) presents the absolute QF values as a function of the inner and outer circle radii. The highest QF (7863) is achieved at an inner radius of 300 nm and an outer radius of 500 nm, while a QF of 5095 is obtained at an inner radius of 300 nm and an outer radius of 600 nm. Figure 6(b) shows the corresponding normalized output power under the same parametric sweep. The maximum normalized output power (97.07) occurs at an inner radius of 200 nm and an outer radius of 600 nm; however, the corresponding QF at this point is relatively low (1388), as seen in Fig. 6(a). Therefore, the final design parameters listed in Table 1 were selected based on a performance trade-off between achieving a high QF and maintaining sufficiently strong normalized output power.

**Fig. 7:**
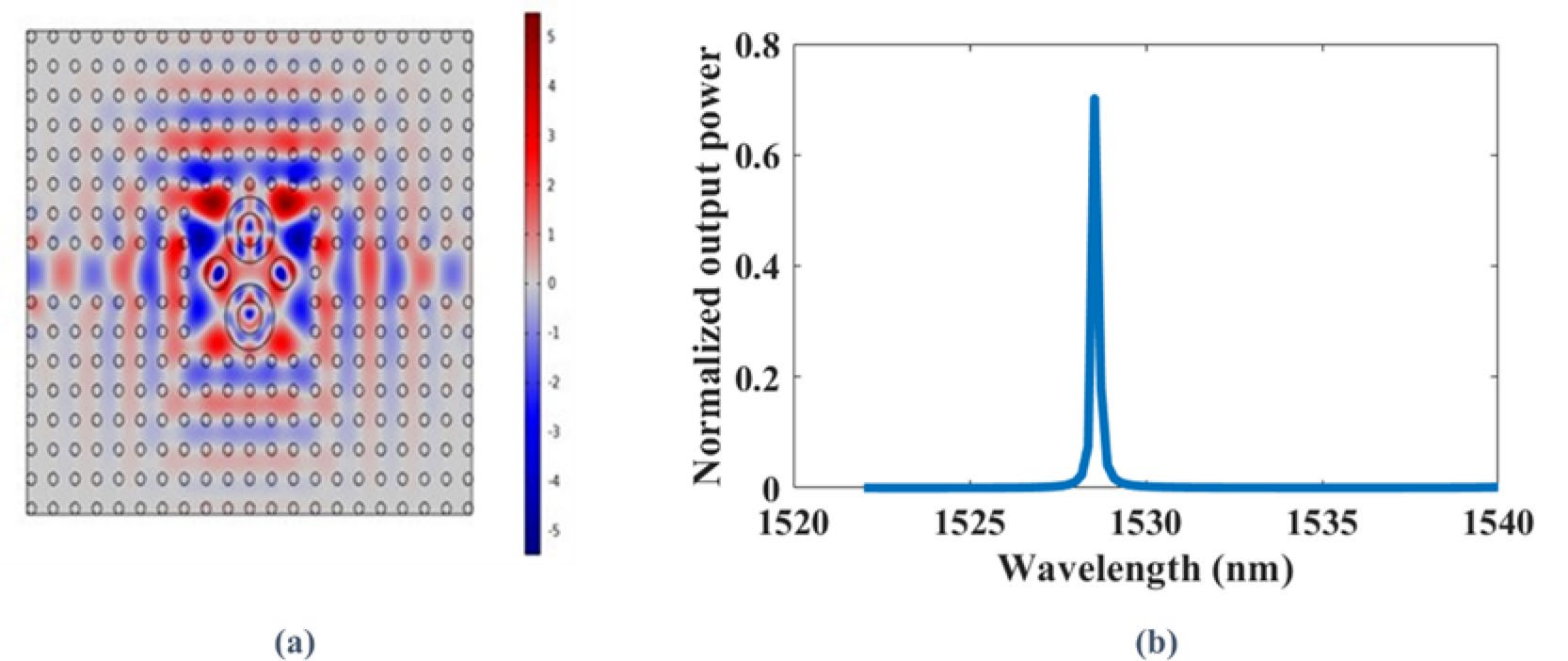
At the optimum chosen geometric parameters for TE mode at λ = 1528.5 nm: **(a)** Electric field distribution, **(b)** Normalized transmission power.

Figure 7(a) shows the electric field distribution when the laser light is guided through the input waveguide and cavities of the optimized 2D nano crystal sensor in TE mode at 1528.5 nm. This confirms that the photonic crystal acts as a mirror on this wavelength that lies within the photonic bandgap feature. The transmission spectrum, in Fig. 7(b), has a transmission efficiency of 70.17% at 1528.5 nm.

Before detecting soil nutrients. Soil preparation must be made. First, the soil from different areas is collected and mixed as one sample^54^. Second, the sample is air-dried and strained through a fine mesh to get rid of rocks, stones, and grasses^55^. Finally, the calcium carbonate is poured, and vinegar is also used since the soil contains alkaline PH to create a saturated solution to get the soil ready for sensing purposes^4^. Following soil preparation, the samples were examined using the proposed 2D-dual-ring Cavity PhC sensor.

**Fig. 8:**
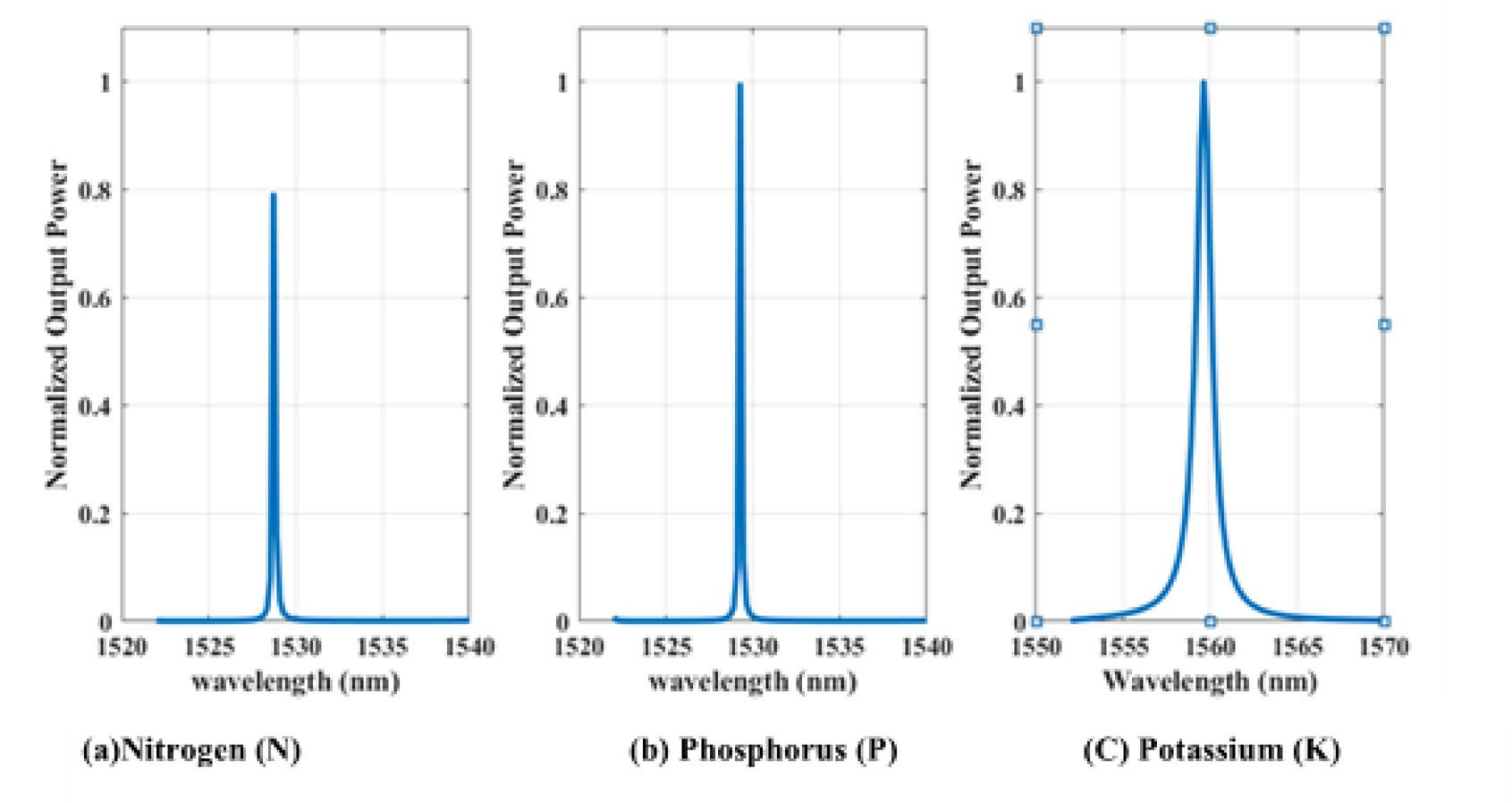
Normalized Transmission spectrum of the proposed photonic sensor for the detection of the most important soil nutrients: **(a)** Nitrogen (N), **(b)** Phosphorus (P), **(c)** Potassium (K).

For clarity, the refractive index values reported in Table 4 do not represent the bulk RI of an aqueous solution. Instead, they are derived from the dielectric constants of individual soil nutrients ($\:n=\surd\:\epsilon\:$) and used as effective electromagnetic parameters within the photonic crystal (PhC) simulation. Since the model operates in an air background (*n* ≈ 1), these values (≈ 1.000–1.002) represent small perturbations caused by each nutrient, not a homogeneous liquid medium. Although real soil solutions are dominated by water (RI ≈ 1.33), the proposed sensor detects relative resonant shifts due to individual nutrients rather than the absolute RI of the bulk solution. Additionally, the sample preparation method is included only to demonstrate practical laboratory implementation and is not used within the simulation model. Accordingly, the resulting spectrum responses of the most important soil nutrients (NPK) are illustrated in Fig. 8.

In this context, it is necessary to distinguish between the sample preparation process and the actual sensing operation. The soil preparation steps described (mixing, air-drying, sieving, and conditioning) are standard procedures intended to ensure controlled and repeatable laboratory evaluation of nutrient concentrations. These preprocessing steps are not part of the sensing time itself. The term “real-time” in this work refers specifically to the rapid optical interrogation and AI-based classification once the sample is introduced into the sensing region. The photonic response and subsequent DCN-v2 inference occur within a short time scale, enabling near-instantaneous detection after measurement initiation. Therefore, while laboratory preparation may require controlled handling, the sensing and decision-making stages operate in real time. Based on this sensing mechanism, the 2D-dual-ring Cavity PhC sensor achieves a high efficiency of 99.39%, a high QF of 18,244, a sensitivity of 631 nm/RIU, FoM of 3440 RIU^− 1^, and a low DoL of 3 × 10^− 5^ RIU as summarized in Table 4.

The nutrient detection results confirm the sensor’s high sensitivity and stability; consequently, the proposed sensor was further employed to assess its potential for microplastics detection in soil. For detecting microplastics, we will focus on low-density polyethylene (LDPE). The soil mixtures can be obtained varying LDPE concentrations (1%,5%,10%,15% by weight) with the refractive index ranging from 2.2 to 2.5^11^.

**Table 4:** The proposed sensing parameters correspond to different soil nutrients.

Nutrient name	RI	$\:{\boldsymbol{\lambda\:}}_{0}$(nm)	QF	S(nm/RIU)	$\:\boldsymbol{\eta\:}$(%)	FoM (RIU^−1^)	DoL (RIU)
Air	1	1528.5	5095	_	70.17	_	_
(N)Nitrogen	1.0003	1528.7	7644	333	79	1665	6 × 10^−5^
(B)Boron	1.00111	1529.3	5098	631	63.76	2103	4.8 × 10^−5^
(P)Phosphorus	1.0012	1529.3	7647	583	99.39	2915	3.4 × 10^−5^
(Zn)Zinc	1.00205	1529.8	7649	585.37	55.31	2927	3.4 × 10^−5^
(k)Potassium	1.33952	1559.6	1418	92	99.94	84	1.2 × 10^−3^
(Ca)Calcium	1.34	1560	1419	93	95.89	85	1.2 × 10^−4^
(Cu)Copper	1.3800	1617.4	5391	234	61.88	780	1.3 × 10^−4^
(Fe)Iron	1.7	1765.3	631	338	89.67	121	8.3 × 10^−4^
(Mn)Manganese	1.8600	1824.4	18,244	344	62.54	3440	3 × 10^−5^

**Table 5:** Proposed sensor response to different LDPE concentrations in soil.

Concentration of LDPE (%)	Refractive index	$\:{{\uplambda\:}}_{0}$(nm)	$\:\eta\:$(%)	QF	S(nm/RIU)	DoL(RIU)	FoM
15	2.2	1847.6	99.8	3695	267	1.9 × 10^− 4^	534
10	2.3	1943.6	70	6479	319	9.4 × 10^− 5^	1063
5	2.4	2074	87.5	2593	390	2.1 × 10^− 4^	488
1	2.5	2176.5	95.48	21,765	432	2.3 × 10^− 5^	4320

Once microplastic soil samples were prepared, the proposed 2D-dual-ring-ring cavity PhC sensor was used to test its ability to detect the LDPE concentrations (1%,5%,10%,15% by weight) as shown in Table 5. It is observed that as LDPE increases from 1% to 15%, the resonance wavelength shifts to longer wavelengths and transmission efficiency, and quality factor change. This shift shows that the proposed 2D-dual- ring cavity PhC sensor is highly sensitive to the surrounding materials, like LDPE microplastics concentrations.

From our knowledge there is a gap in the detection of soil nutrients utilizing photonic crystal-based sensors. Table 6 presents a comparison of the proposed structure with similar structures found in the literature. The QF, S, FoM and DoL of the proposed sensor is markedly improved relative to those of Ref^4^ in terms of soil nutrient detection. In this context, as shown in Table 6, the detection limit (DoL) values for all sensors are calculated using Eq. (5), ensuring a consistent comparison. While some reported designs achieve lower DoL values, the proposed dual-ring cavity sensor demonstrates a comparatively high Q-factor and competitive sensing performance among 2D photonic crystal platforms.

**Table 6:** Comparison between the proposed sensor and previously reported designs.

Ref.	Sensor type	Target	QF	S (nm/RIU)	DoL (RIU)	FoM(RIU^− 1^)
^40^	1D PhC	hazardous gases	300,000	700	*8.3 × 10^− 6^	149,000
^41^	2D PhC	bacteria in water	*10,000	834.344	*1.2 × 10^− 5^	* 1668.7
^36^	1D PhC	Blood plasma and cancer cells	19.079	72.5	* 0.00135	*20
^56^	PhC fiber	benzene, chloroform, ethanol and water	*850	*7000	*1.25 × 10^− 5^	*1600
^38^	2D PhC	Pressure	772	*2000	*5 × 10^− 6^	*1000
^39^	Ternary PhC	water concentration in ethanol solution	8390.28	144.3	0.00247524	6633.55
^33^	2D PhC	Brain tumor	1332	1645	9.08 × 10⁻⁶	*11,000
^4^	Photonic crystal	Soil nutrients	228.9	334	*3.4 × 10^− 5^	*49
Our 2D-dual-ring cavity PhC sensor	2D PhC	Soil nutrients	18,244	631	3 × 10^− 5^	3440
Our 2D-dual-ring cavity PhC sensor	2D PhC	LDPE in soil	21,765	432	2.3 × 10^− 5^	4320

* Estimated from the data reported in the referenced publications.

### Stage 2: The detection performance of the deep model

**Fig. 9:**
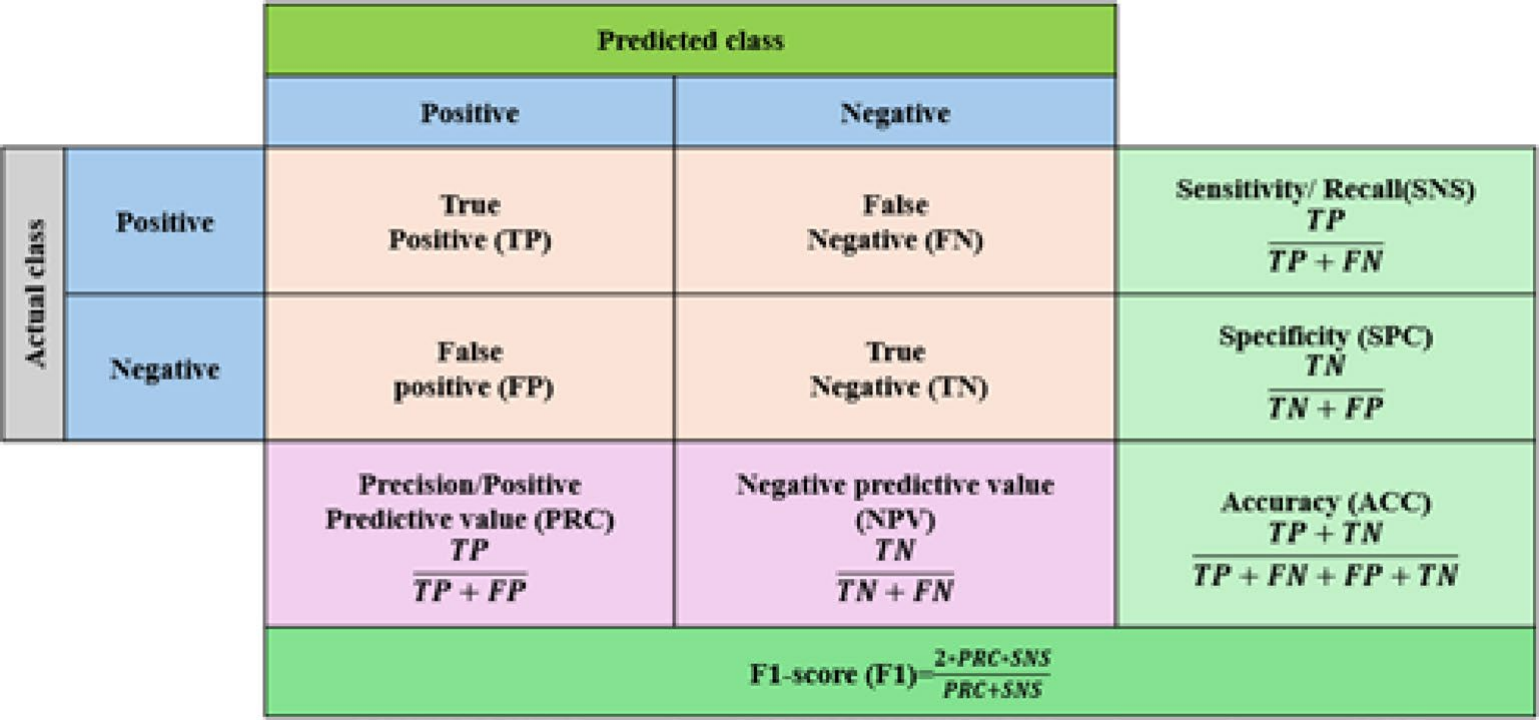
Confusion matrix showing performance metrices formula.

The classification model was trained to identify ten soil-related classes comprising nine essential plant nutrients (B, Ca, Cu, Fe, Mn, N, P, K, Zn) and one soil contaminant (LDPE). The nutrient classes represent both primary macronutrients (N, P, K), secondary macronutrients (Ca), and key micronutrients (B, Cu, Fe, Mn, Zn). All these types play critical roles in plant growth and soil fertility. In contrast, LDPE represents a non-biodegradable plastic contaminant commonly found in agricultural soils due to much film degradation and plastic waste accumulation. The dataset was split to 64%−16%−20% for training, validation, and testing, respectively. All experiments were performed on the Colab Pro environment of Google using different Python libraries, such as Keras, NumPy, Tensorflow, and Sklearn. We set the learning rate parameter to 0.0001. Adam is used as optimizer, and categorical cross entropy is employed as a loss function. Finally, the model is trained with batches of 32 due to memory limitations. For the model assessment, different metrices are employed, such as accuracy, precision, recall, and F1-score. These metrices are summarized in Fig. 9.

**Fig. 10:**
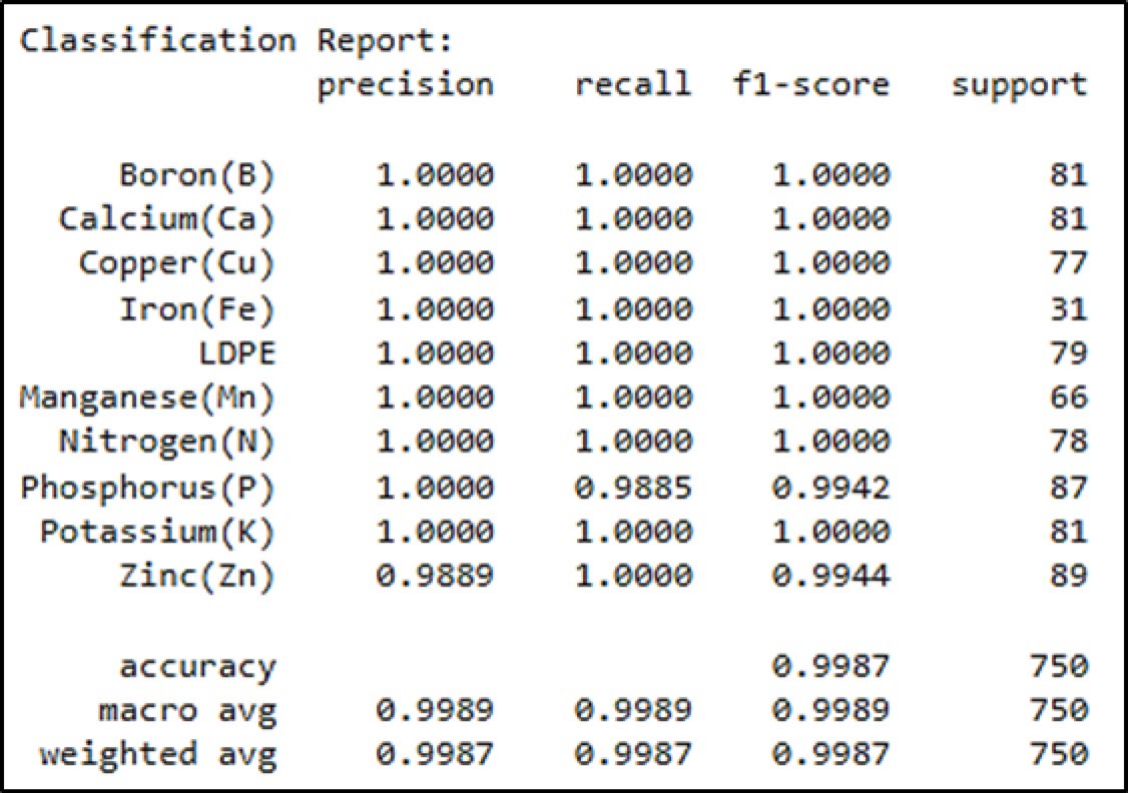
Classification report of the testing part of the dataset.

**Fig. 11:**
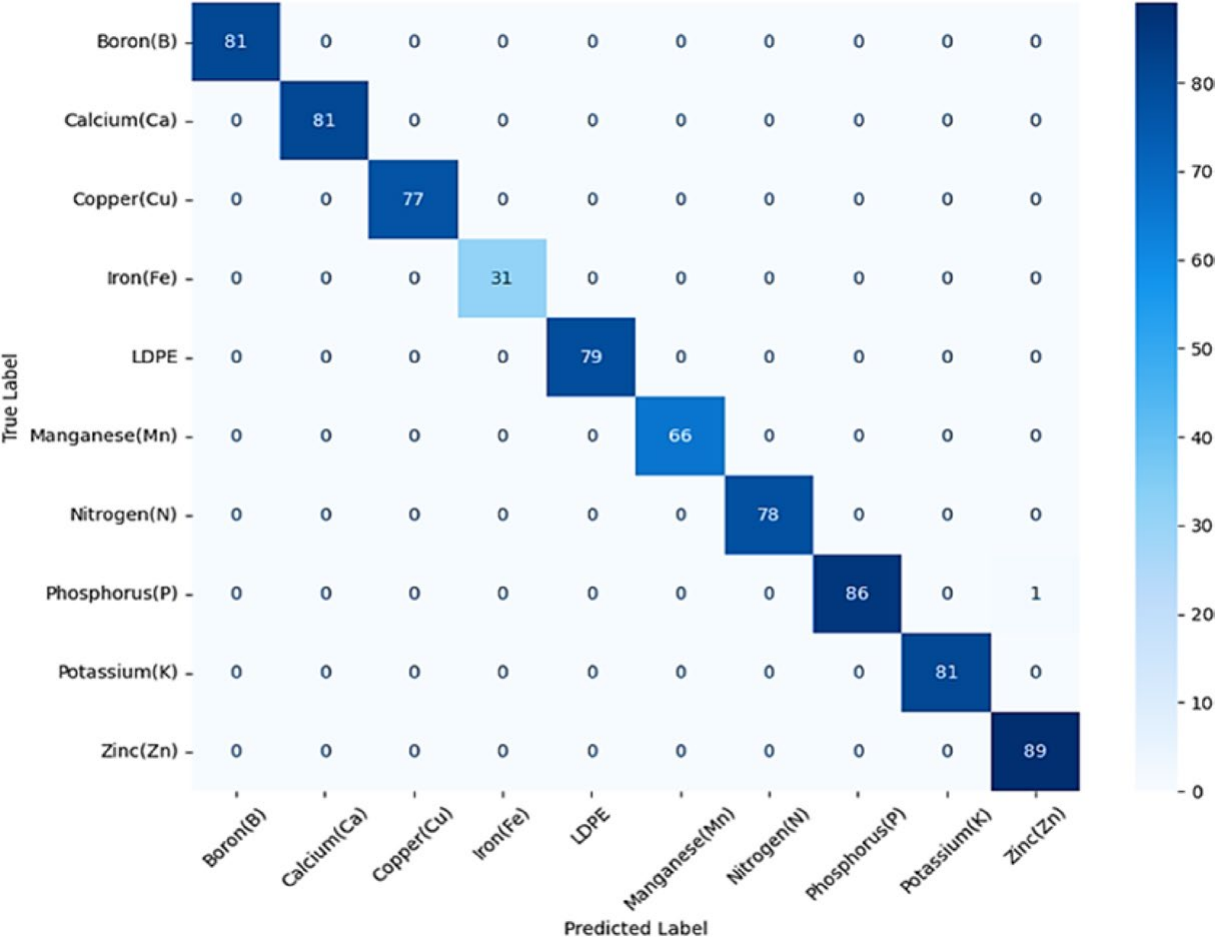
Confusion matrix of the testing part of the dataset.

Figures 10 and 11 indicate the classification report and confusion matrix of the testing part of the dataset, respectively. The classification model achieved exceptionally high accuracy (99.87%) in identifying both soil nutrients and soil contaminants. This shows the model’s ability not only to differentiate between various nutrient types but also to reliably detect contaminant material within soil samples.

**Fig. 12:**
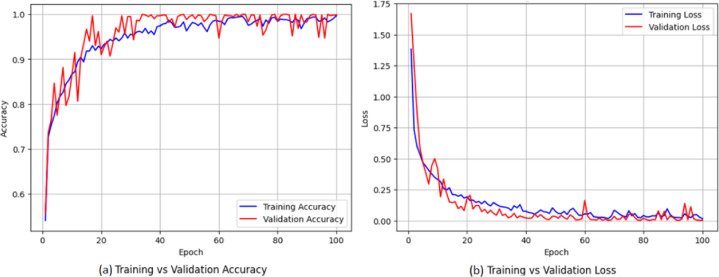
**(a)** the training-validation performance across epochs in terms of accuracy, **(b)** the training-validation performance across epochs in terms of loss.

**Fig. 13:**
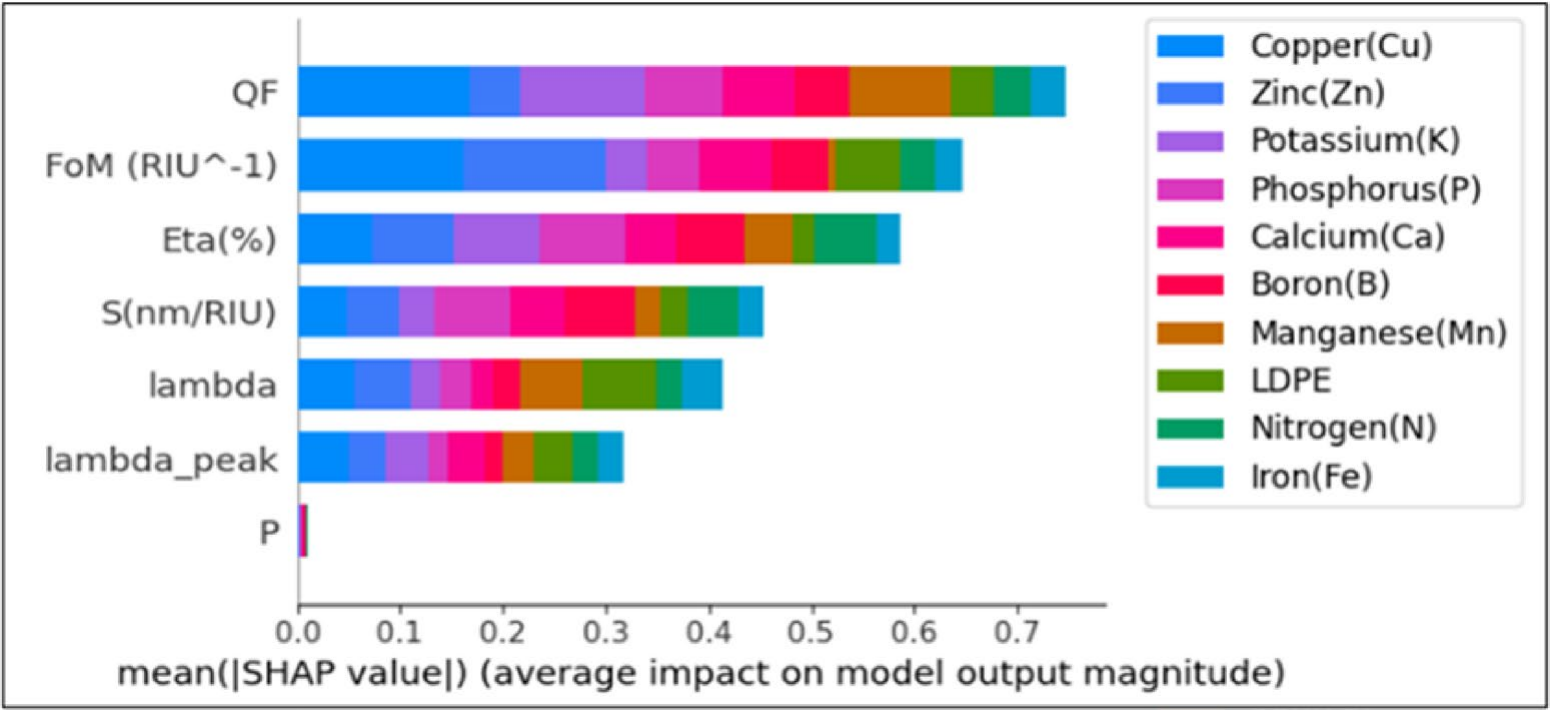
Mean absolute SHAP values for all features for all nutrients and contaminants.

In Figs. 12 (a), (b) the training-validation performance is indicated over epochs in terms of accuracy and loss. To enhance the interpretability of the Deep Cross Network v2 (DCN-v2) model used for multiclass classification of ten soil elements, we applied two widely used model-agnostic explainability techniques: SHAP [51] and LIME [52]. SHAP (Shapley Additive explanations) quantifies the contribution of each input feature to the model’s output using principles from cooperative game theory. Hence, it provides a consistent and theoretically grounded measure of global and local feature importance across all soil-element classes. LIME (Local Interpretable Model-Agnostic Explanations) offers complementary insights by generating local surrogate models around individual predictions. Thus, it allows understanding why a specific element label is assigned to a given soil sample. By combining SHAP’s holistic importance analysis with LIME’s instance-level interpretability, we gain a comprehensive understanding of the DCN-v2 decision process, thereby increasing transparency and trust in the PhC sensor–based soil-element detection pipeline. To quantify the relative influence of each input feature on the model predictions, we computed the mean absolute SHAP values for all features in Fig. 13. The plot ranks the features according to their overall contribution to the model output. Quality factor (QF) and figure of merit FoM (RIU⁻¹) exhibited the highest mean |SHAP| values, indicating that they have the strongest impact on the predictive behavior of the model. These are followed by power efficiency (η) and Sensitivity (nm/RIU). Both wavelength (λ) and peak wavelength (λ_peak) demonstrate moderate importance, whereas normalized power (p) shows negligible impact.

**Fig. 14:**
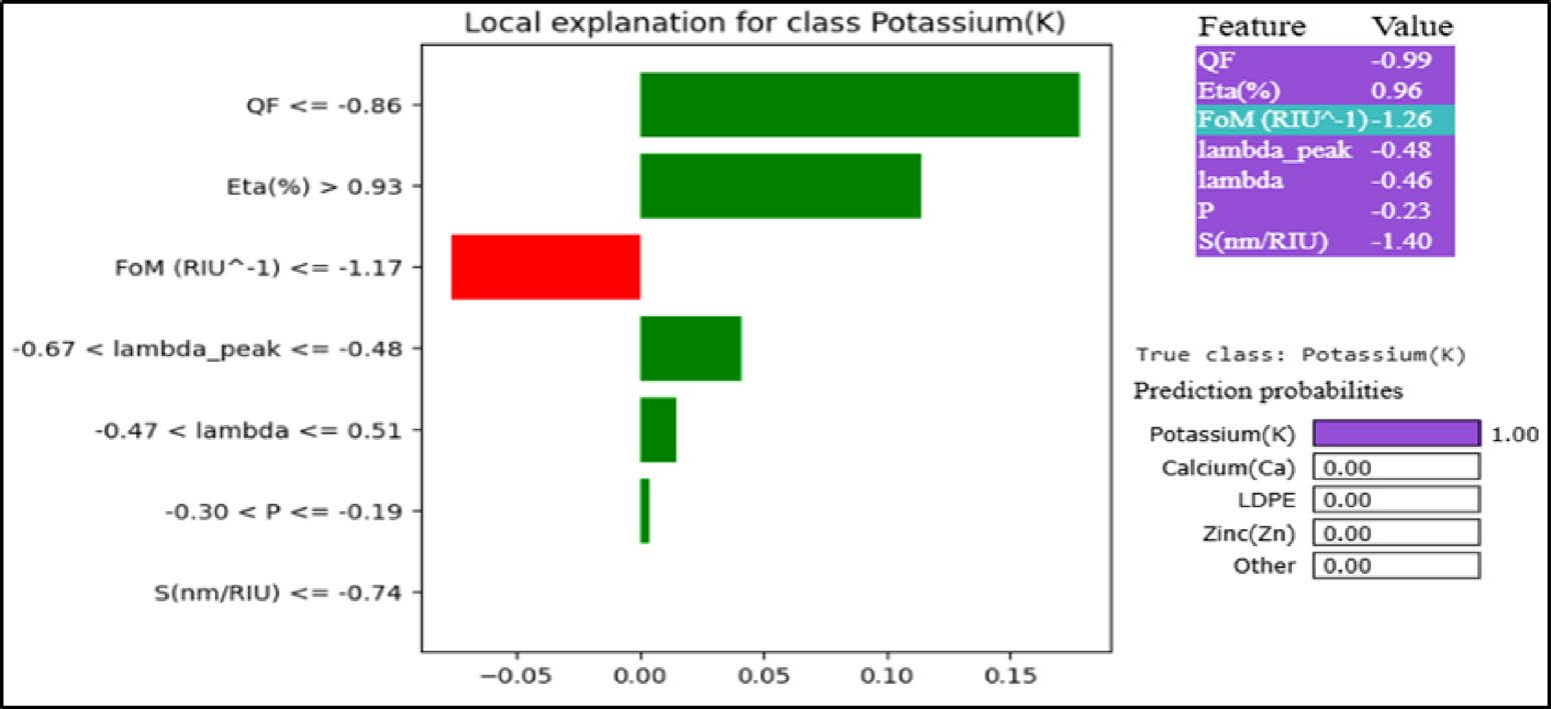
LIME-based local explanation for Potassium (K) (index = 51).

Figure 14 presents the LIME-based local explanation for a representative test sample (index=51) that the model classified as Potassium (K) with a predicted probability of 1.00. The Lime analysis role is to identify the specific feature contributions that locally influenced the model’s decision. LIME approximates the behavior of the DCN-v2 classifier around the selected instance using an interpretable linear model. Each bar in the indicated figure demonstrates the direction and magnitude of a feature’s influence on the prediction for Potassium (K). The green bars represent positive contributions and push the prediction toward the Potassium class, while the red bars represent negative contributions and push the prediction away from it. The most influential features supporting the Potassium (K) prediction are QF ≤ −0.86 and Eta (η) (%) > 0.93, which provide the strongest positive contributions toward the final classification. Additional features such as −0.67 < λ_peak ≤ −0.48, −0.47 < λ ≤ 0.51, and −0.30 < *P* ≤ −0.19 also contribute positively, although their effects are comparatively smaller and act mainly as supportive evidence consistent with the model’s learned decision boundaries. In contrast, FoM (RIU⁻¹) ≤ −1.17 is the only feature exhibiting a negative contribution, slightly pushing the prediction away from the Potassium class. However, the magnitude of this negative effect is limited and cannot offset the strong positive influence of QF and efficiency. The feature S (nm/RIU) ≤ −0.74 shows a negligible impact in this specific instance, indicating that it plays only a minor role in the local decision. Overall, the local explanation demonstrates that the model’s prediction is primarily driven by the combination of low QF and high efficiency, supported by consistent wavelength and pressure conditions. The dominance of positive contributions explains the model’s extremely high confidence (probability ≈ 1.00) in assigning this sample to the Potassium class.

Table 7 summarizes the performance of the proposed DCNv2 classifier against a wide range of classical machine-learning models and neural baselines in terms of different evaluation metrics, including accuracy, macro-precision, macro-recall, macro–F1 score, and macro-ROC–AUC. As indicated in the comparative results, DCN-v2 achieves the highest performance across all evaluation metrics, reaching an accuracy of 0.9987, macro-precision of 0.9988, macro-recall of 0.9989, macro-F1 of 0.9988, and a perfect ROC–AUC of 1.0000. Among the classical machine learning approaches, Gaussian Naïve Bayes (GNB) demonstrates notably strong performance (Accuracy of 0.9307; F1 of 0.9376), outperforming several traditional classifiers including Logistic Regression, SVM variants, LDA, and tree-based models. The strong performance of GNB suggests that class distributions are relatively separable and partially consistent with probabilistic assumptions. Logistic Regression CV also provides competitive results (Accuracy of 0.9133; F1 of 0.9207), indicating that optimized linear models can capture a significant portion of the discriminative structure. Kernel-based approaches such as SVM (RBF and polynomial) achieve moderately strong performance (Accuracy ranging from 0.7453 to 0.8320), reflecting their ability to learn nonlinear decision boundaries. Neural network models further improve performance; the Small ANN achieves an accuracy of 0.8840 and F1 of 0.8931, while the deeper ANN architecture (128–64–32) significantly enhances performance (Accuracy of 0.9760; F1 of 0.9785), highlighting the benefit of deeper feature representation learning. Ensemble methods such as Gradient Boosting also perform strongly (Accuracy of 0.9667; F1 of 0.9699), demonstrating the effectiveness of nonlinear hierarchical learning. In contrast, classifiers such as Ridge Classifier, Bernoulli NB, Nearest Centroid, and LDA show comparatively lower performance, likely due to their linear assumptions or limited capacity to model complex nonlinear relationships within the dataset. Overall, DCN-v2 consistently outperforms all classical and deep learning baselines, demonstrating a superior ability to model complex feature interactions and achieve extremely high classification confidence and robustness across all evaluation metrics.

**Table 7:** Comparison between the proposed DCNv2 classifier and other classical machine learning models.

Classifier	Accuracy	Precision (Macro)	Recall (Macro)	F1-score (Macro)	ROC-AUC (Macro)
Logistic Regression	0.7053	0.6995	0.7413	0.7158	0.9497
Logistic Regression CV	0.9133	0.9225	0.9232	0.9207	0.9922
Ridge Classifier	0.5547	0.4973	0.5391	0.4821	nan
SVM (RBF)	0.8320	0.8521	0.8534	0.8467	0.9799
SVM (Poly)	0.7453	0.8160	0.7777	0.7644	0.9762
Gaussian NB	0.9307	0.9372	0.9380	0.9376	0.9921
Bernoulli NB	0.6040	0.5743	0.6187	0.5809	0.9336
LDA	0.5987	0.6031	0.6419	0.6051	0.9464
Nearest Centroid	0.5387	0.5427	0.5876	0.5316	0.9368
Small ANN	0.8840	0.8951	0.8980	0.8931	0.9924
Decision Tree	0.7240	0.6281	0.7000	0.6439	0.9364
Gradient Boosting	0.9667	0.9764	0.9713	0.9699	0.9963
Deep ANN (128-64-32)	0.9760	0.9827	0.9779	0.9785	1.0000
DCN-v2	0.9987	0.9988	0.9989	0.9988	1.0000

### Field deployment and environmental robustness analysis

For practical smart agriculture applications, the proposed photonic–AI sensing framework does not require bulky laboratory instruments, as the resonance-shift-based response can be interrogated using compact optical readout units such as miniaturized spectrometers or wavelength-selective photodetector systems with low-power laser sources. Moreover, the trained DCN-v2 model can be deployed on embedded processors for real-time classification, enabling a portable and potentially field-deployable sensing platform.

**Fig. 15:**
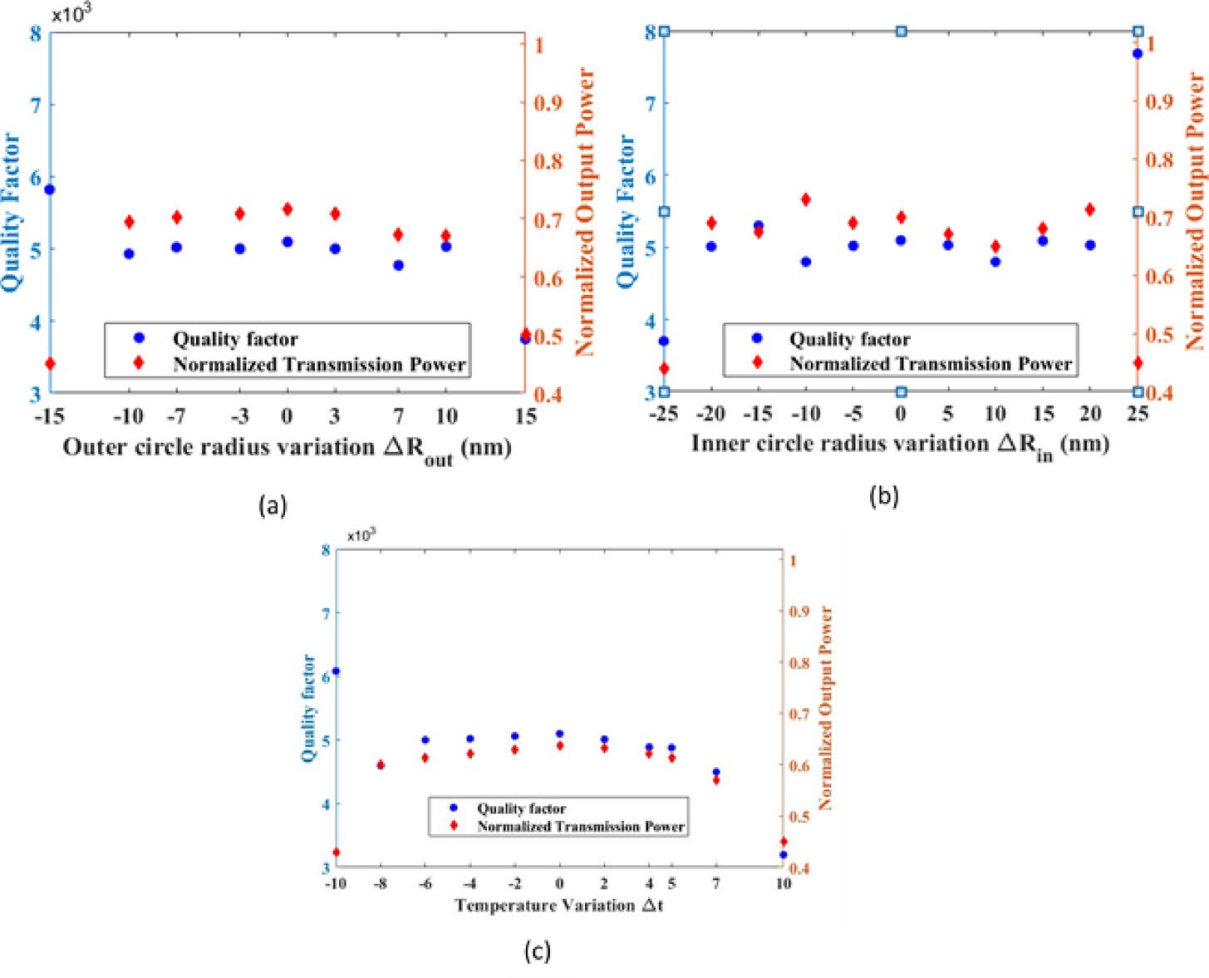
Fabrication tolerance analysis: **(a)** variation of the outer silicon cavity radius, **(b)** variation of the inner germanium cavity radius, and **(c)** temperature variations near room temperature (25 °C) for boron nutrient detection.

To assess the robustness of the proposed sensor under practical operating conditions, Fig. 15 Shows the influence of fabrication tolerances and environmental temperature variations. Key geometric parameters of the structure were varied to account for fabrication uncertainties, including deviations of ± 10 nm and ± 20 nm in the outer radius of the silicon cavity and the inner radius of the germanium cavity, respectively. In addition, the sensor response was evaluated under temperature variations of ± 5 °C around room temperature (25 °C). Within these investigated ranges, the sensor exhibits stable transmission characteristics and consistent quality-factor behavior, indicating reliable measurement performance. Although a full probabilistic robustness analysis involving extensive parameter distributions would require a significantly larger number of simulations and substantial computational effort, the present results provide initial confidence in the robustness and practical viability of the proposed design.

While the sensor exhibits stable performance under fabrication tolerances, the feasibility of experimentally realizing the concentric silicon–germanium cavity design using established fabrication processes is an important practical consideration. Advanced nanofabrication methods are needed to ensure the required geometric precision and alignment for the fabrication of the proposed sensor. The recommended fabrication methods include electron beam lithography (EBL)^57^, deep ultraviolet lithography (DUV)^58^, reactive ion etching (RIE)^59^, deep reactive ion etching (DRIE)^60^ and focused ion beam (FIB) milling^61^.

These high-resolution methods allow for exceptional precision in sub-wavelength structures. The fabrication process^62^ starts with silicon rods as the core material, then obtaining the inner Germanium cavity. Electron beam lithography is suitable for this stage for its nanometer-scale precision, followed by (RIE) producing a smaller concentric cylindrical. The two techniques are used for obtaining the outer silicon cavity. Since the sensor works in the near-infrared region, the material platform is completely compatible with silicon photonics and optical integration technology. This allows for the possibility of integration with waveguides, optical fibers, or lab-on-a-chip sensing platforms. However, the creation of concentric silicon–germanium cavity structure may pose some difficulties. For instance, (EPL) could be affected by proximity effects, particularly in the case of a curved boundary of the cavity. This could cause line edge roughness, which in turn can cause scattering losses^63^. Moreover, (RIE) could cause sidewall tapering^64^. To overcome the limitations of high-resolution nanofabrication, several advanced strategies have been developed such as Proximity Effect Correction (PEC)^63^ and focused ion beam (FIB) milling^65^ are used to enhance fabrication accuracy for complex photonic structures.

The realization of the proposed concentric silicon–germanium photonic crystal cavity relies on established hybrid material processing, including silicon–germanium integration and nanoscale pattern definition, as well as compatibility with on-chip photonic and electronic platforms. In addition, implementation of the DCN-v2 model requires integration with an embedded processing unit or dedicated AI-capable chip to enable real-time inference within a compact sensing module. Such requirements are intrinsic to advanced photonic crystal sensor fabrication and align with standard practices in integrated photonic and intelligent sensing system development.

## Conclusion

This study introduces a fully simulation-driven photonic–AI sensing framework for advanced soil nutrient and microplastic detection. The proposed approach forms an integrated end-to-end smart soil sensing platform that combines a dual-ring photonic crystal cavity sensor with a Deep & Cross Network architecture. The designed PhC sensor exhibits strong light confinement and high spectral discrimination, delivering high quality factors, excellent sensitivity for both nutrient elements and LDPE microplastics, low detection limits, and competitive figures of merit. Fabrication tolerance and temperature variation analyses further demonstrate stable and reliable performance under practical structural deviations and moderate environmental fluctuations. From DCN side, it learns complex nonlinear relationships within the spectral responses without explicitly incorporating refractive index as an input parameter. Hence, the ambiguities among closely related concentration levels that cause very close refractive indices are minimized. The framework achieves near-perfect classification metrics, while SHAP and LIME analyses validate the physical significance of the most influential spectral features, strengthening model transparency and reliability. Hence, the integration of optimized photonic sensing and explainable deep learning establishes a compact, automated, and high-accuracy solution with strong potential for next-generation smart agriculture and environmental monitoring applications. However, this work is currently simulation-based, so real soil conditions, like variability in moisture, composition, and noise, may introduce additional factors to consider. Accordingly, next steps will include experimental testing, collecting real soil data, expanding robustness analyses, and building an integrated prototype with the photonic sensor, compact optical readout, and embedded AI. These efforts will help translate our framework into a practical, field-ready solution for smart soil monitoring.

## Data Availability

Data will be made available on request.
